# Luminescent Probe Based on Terbium-Carbon Quantum Dots for the Quantification of Imidacloprid in Caneberries

**DOI:** 10.1155/2023/5561071

**Published:** 2023-10-30

**Authors:** Eulogio J. Llorent-Martínez, Julia Jiménez-López, Antonio Ruiz-Medina

**Affiliations:** Department of Physical and Analytical Chemistry, Faculty of Experimental Sciences, University of Jaén, Campus Las Lagunillas, E-23071, Jaén, Spain

## Abstract

We propose a modification of terbium-sensitized luminescence (TSL) by means of the introduction of nanoparticles to improve the sensitivity and selectivity of the analytical methods. TSL detection is usually based on the complexation between fluorescent organic compounds (the analytes) and terbium. The organic compound is then excited, and, after an energy transfer towards terbium, the latter emits the luminescence signal. Here, the modification consists of the introduction of nanoparticles (carbon quantum dots, CQDs) into the system. The carboxylic groups of CQDs react with terbium, providing an interesting time-resolved luminescence probe. We applied this system for the determination of the neonicotinoid imidacloprid (IMID). When IMID was introduced in the terbium-CQDs system, the luminescent signal (*λ*_exc_/*λ*_em_ of 256/545 nm) was quenched, proportionally to IMID concentration in the range of 100–2500 ng·mL^−1^, obtaining a limit of detection of 30 ng·mL^−1^. A method detection limit of 0.9 mg·kg^−1^ was reached in caneberries, thus complying with the maximum residue level of 5 mg·kg^−1^ established by Codex Alimentarius. We performed recovery experiments in caneberries (blackberries, blueberries, raspberries, and mulberries), obtaining recovery yields close to 100% in all cases. These results show that the use of terbium ions-nanoparticles luminescence probes can be useful for screening purposes in quality control laboratories.

## 1. Introduction

Lanthanide-sensitized luminescence (LSL) is a detection technique that presents interesting spectroscopic properties for its successful use in the development of analytical methods of analysis. The main advantages of LSL (using both terbium or europium ions) are the high Stokes shift and the long lifetimes of the excited states, which permits using time-resolved luminescence, hence eliminating any fluorescence background interferences [1]. In conventional LSL, lanthanide ions form a complex with the analyte (usually, a fluorescent organic compound). Then, the excitation energy is absorbed by the analyte and transferred to the excited state of the lanthanide, which emits luminescence. As a result, the excitation and emission wavelengths correspond to the analyte and lanthanide ions, respectively. However, LSL also suffers from some disadvantages, mainly the need for rigidity in the complex and the requirement of avoiding the presence of water molecules, which can quench the analytical signal. It is thus required to use surfactants and synergistic agents, which are in contrast to the principles of green chemistry, to improve LSL sensitivity [2, 3].

A recent approach to improve the characteristics of LSL-based analytical methods is the use of nanoparticles (NPs). In this sense, silver and gold NPs have been used [4, 5]. The interaction of any of these two NPs with lanthanide ions can be explained through metal-enhanced fluorescence (MEF), which results from the coupled interaction between the fluorophore (specifically, its electrons) and the plasmon resonance of metal NPs (surface plasmon). In this way, MEF produces an increase in the fluorescence emission and the radiative decay of the system [6]. Another alternative to these NPs is the use of carbon-based quantum dots. Among them, carbon quantum dots (CQDs) as well as graphene quantum dots (GQDs) both have acquired great interest in the scientific world due to their unique physical and chemical properties, which are caused by quantum confinement and edge effects [7]. Moreover, they use raw materials that can be easily obtained; they have interesting characteristics, such as their bioconjugation and ease of synthesis. Carbon-based quantum dots can compete with semiconductor quantum dots because they present similar electronic and physicochemical properties but avoid the high toxicity of semiconductor quantum dots. Moreover, they can be functionalized using different molecules of organic, inorganic, and biological nature and even polymers or natural compounds. For this reason, they have gained much attention in biomedical and bioanalytical applications. In recent years, different systems have been proposed by coupling GQDs and LSL [8, 9], where GQDs form complexes with lanthanide ions through the carboxylate functional groups of the NPs, thereby increasing the luminescence of lanthanide. However, the coupling of LSL and CQDs is still practically unexplored [10]. Although GQDs and CQDs are very similar with respect to their optoelectronic properties, the main advantages of CQDs are that they are simpler and cheaper to obtain (both commercially or synthesized in the laboratory).

Neonicotinoids have been extensively used worldwide as insecticides to treat soil and seeds. However, several studies have mentioned the harm caused to bees and, therefore, to entire ecosystems. The Joint FAO/WHO Meeting on Pesticide Residues have reported that the neonicotinoids imidacloprid, thiamethoxam, and clothianidin were the most likely ones to remain in plant commodities and their processed products after physicochemical and biological processes [11]. For this reason, the European Union banned the use of these compounds in 2018, although today there are many countries on other continents that are still using them [12]. Therefore, it is crucial to develop easy, fast, and efficient methods to quantitatively determine neonicotinoid pesticide residues. We have selected imidacloprid (IMID) as the target compound in this work. The main analytical methods for its analysis in food samples involve liquid chromatography [13–16]; nevertheless, despite their high detection accuracy and precision, these methods require complex and time-consuming laboratory procedures. UV/vis absorption [17], Raman [18, 19], chemiluminescence [20, 21], or fluorescence [22–25] spectroscopy are also alternatives for IMID determination.

This work aims to develop an analytical method for the determination of IMID in caneberries, a type of berry fruit, based on the modification of terbium-sensitized luminescence (TSL) by CQDs. The luminescence of the Tb(III)-CQDs system is quenched by IMID, thus allowing its quantification. This approach is in contrast with the typical increase of TSL signal by the complexation with the analyte. However, this alternative has proved successful for the analysis of a nonfluorescent analyte such as IMID, as the method detection limit (MDL) achieved in this work was satisfactory to comply with the maximum residue limit (MRL) of 5 mg·kg^−1^ established by Codex Alimentarius in berry fruits [26]. A modified QuEChERS approach was used as sample pretreatment. The results of the present study might serve as a useful means for regulatory organs concerning food quality control.

## 2. Experimental

### 2.1. Instrumentation

**Figure 1:**
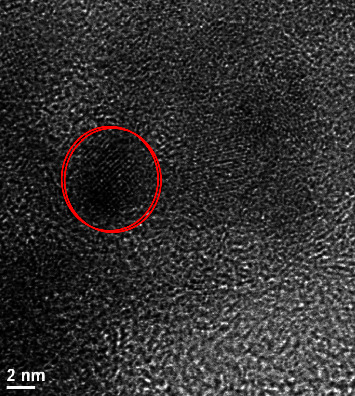
TEM image of CQDs.

A Cary Eclipse luminescence spectrometer (Varian, Mulgrave, Australia) with a Hellma 1010-QS cell (3500-*μ*L volume and 10 mm × 10 mm light path) was used to record luminescent signals. A high-resolution transmission electron microscopy using an FEI Titan (FEI Europe B.V., Eindhoven, Netherlands) at 300 kV was utilized to study the morphology of CQDs (Figure 1). The used instrument has an Image CS corrected; digital images were captured with a camera Gatan model GIF2002P with image processing. The samples were prepared by depositing an aqueous solution onto a copper carbon film. It allowed a magnification range of 500x up to 400000x.

### 2.2. Reagents and Solutions

Sodium hydroxide (≥99%), sodium acetate 3-hydrate (≥99%), tris(hydroxymethyl)aminomethane (99.5%), and hydrochloric acid (37%) were obtained from Panreac (Barcelona, Spain). CQDs, acetonitrile (99.8%), sodium borohydride (98%), sodium phosphate dibasic (99.9%), sodium dihydrogen phosphate (≥99%), graphitized carbon black (GCB), Eu (III) nitrate pentahydrate (99.9%), Tb (III) nitrate pentahydrate, and interferents (malathion, glyphosate, chlorpyrifos, acetamiprid lambda-cyhalothrin, Ca^2+^, K^+^, Mg^2+^, and Na^+^) were purchased from Merck (Madrid, Spain). ISOLUTE QuEChERS kits (extraction and clean-up) were bought from Biotage (Sweden).

Ultrapure water (Milli-Q water purification system; Millipore; Milford, MA) was used in all experiments.

IMID (≥98%) was obtained from Merck. A stock solution of 20 mg·L^−1^ was prepared in ultrapure water. Stock solutions of Tb (III) (0.1 mol·L^−1^), Eu (III) (0.02 mol·L^−1^), and CQDs (100 mg·L^−1^) were prepared in ultrapure water. All solutions were kept in the fridge, protected from light, and were stable for at least 3 months. Suitable dilutions were made in ultrapure water when required.

### 2.3. General Procedure

All solutions were prepared in centrifuge tubes, completing to 2 mL volume with ultrapure water (previously adjusted to pH 6 with HCl). The following solutions were used: 30 *μ*L Tb (III) (0.1 mol·L^−1^), 200 *μ*L CQDs (100 mg·L^−1^), and the required volume of IMID (20 mg·L^−1^) to obtain IMID concentrations in the range of 100–2500 ng·mL^−1^. The final concentrations of Tb (III) and CQDs were 1.5 × 10^–3^ mol·L^−1^ and 10 mg·L^−1^, respectively.

After shaking for 10 s, the solution was transferred to the 1-cm quartz cell and the emission spectrum was recorded at an excitation wavelength of 256 nm after an incubation time of 15 min. The optimum instrumental conditions were excitation/emission slit widths of 10/10 nm/nm, delay and gate time of 0.1 ms and 3 ms, respectively, and photomultiplier tube voltage of 780 V.

Peak heights at 545 nm were measured for standard and sample solutions. The net analytical signal was the signal of Tb (III)-CQDs minus the signal of Tb (III)-CQDs-IMID; that is, the analytical signal used was the quenching produced by IMID in the original Tb (III)-CQDs signal.

### 2.4. Sample Treatment

In foodstuffs, a sample preparation step is needed to eliminate or reduce the matrix effects. Among these methods, it is worth mentioning different approaches for the extraction of neonicotinoid insecticides: liquid-liquid extraction [27], liquid-phase microextraction [28], dispersive liquid-liquid microextraction [29], solid-phase extraction [30], and magnetic solid-phase extraction [31]. However, these methods present one or more of the following disadvantages: large organic solvent consumption, low adsorption capacity of the sorbent, high cost, or low recoveries [32]. The modified QuEChERS method is considered to be an optimal sample treatment for the simultaneous determination of multiple pesticide residues [33], including IMID, so it was the selected one.

A total of ten samples were analyzed: three different trademarks of blackberries and raspberries and two trademarks of blueberries and mulberries. All of them were purchased at local markets. Approximately 50 g of each sample was thoroughly ground and homogenized. A QuEChERS extraction adapted from [34] was used. Specifically, a QuEChERS extraction kit (ISOLUTE QuEChERS) with GCB was used for all the samples. GCB was required due to the high pigmentation of the samples. Otherwise, lower recovery yields were obtained in the analyzed berries (recovery experiments are detailed in Section 3.6). Sample treatment is detailed in Supplementary Materials (available here).

## 3. Results and Discussion

### 3.1. Preliminary Studies and Proposed Mechanism

First, the luminescence spectra of CQDs were recorded, observing maximum excitation/emission wavelengths at 345/445 nm/nm. Then, the study of the direct interaction between CQDs and IMID was tested, but no changes in the fluorescence spectra were observed (without the use of lanthanide ions, only fluorescence could be tested and not time-resolved luminescence). The addition of lanthanide ions was thus required in the system.

As mentioned before, LSL is employed for the determination of fluorescent analytes. As IMID does not present native fluorescence, a different approach needs to be considered, that is, its quenching effect on the luminescence system. IMID produced a decrease in the time-resolved luminescence of both Tb (III) and Eu (III). This effect was previously reported by our research group [35, 36]. Therefore, we tested the effect produced in the system by the introduction of CQDs. When CQDs were used along with the lanthanide ion, an increase in sensitivity of around 30–40% was obtained. The use of Tb (III)-CQDs instead of Eu (III)-CQDs resulted in a slight improvement in sensitivity (10%). As a result, we selected Tb (III)-CQDs as the optimum luminescence probe for further studies with IMID. In fact, terbium ions usually produce higher sensitivity than europium ions in LSL-based analytical methods.

The interaction between Tb (III) and CQDs is probably due to the formation of a complex with luminescent properties through the carboxylic groups present in the CQDs. In LSL, synergetic agents or surfactants are frequently used to exclude water, which is a potential quencher in this type of a system. However, alternative approaches can be implemented to avoid the use of additional reagents and thus achieve more environmentally friendly methods. In this work, the use of CQDs provides stability to Tb (III) ions since these NPs allow the exclusion of water molecules, thus increasing the sensitivity of the system. An advantage of the selected NPs is the possibility of being able to acquire them commercially for achieving a better reproducibility between different laboratories (problems may arise in precision when using different apparatus or instrumentation) and avoiding laborious syntheses.

The potential quenching mechanism of the proposed system was evaluated by the Stern–Volmer equation. The plot is shown in Figure S1 (Supplementary Material). The relationship between *I*_0_/*I* and *Q* was linear, where *Q* is the quencher concentration (IMID concentration), *I*_0_ is the luminescence signal without the presence of the quencher, and *I* is the luminescence signal with the presence of the quencher. The obtained value of the Stern–Volmer constant (K_SV_) was 0.1759 *μ*g·mL^−1^. The plausible mechanism, in this case, is a collisional quenching where the analyte interacts with the Tb (III)-CQDs system. IMID breaks the complex previously formed between both the components.

### 3.2. Instrumental Variables

Excitation and emission spectra of the Tb(III)-CQDs system were recorded (using time-resolved luminescence), obtaining maximum excitation/emission wavelengths of 256/545 nm/nm, which correspond to Tb (III). This emission wavelength corresponded to the Tb (III) ^5^D_4_ ⟶ ^7^F_5_ transition, which is the most sensitive of Tb (III). Hence, the introduction of CQDs provides stability and rigidity to the system, thereby increasing the luminescence signal, but it does not change the time-resolved luminescence signals. As Tb (III) presents three emission bands (490, 545, and 590 nm), the whole emission spectrum was recorded with and without the presence of IMID. However, the peak at 545 nm provided the highest analytical signal (highest IMID quenching) and was the selected one for further experiments.

By using the optimum wavelengths, the following instrumental parameters were optimized: delay time (0.1–0.3 ms), gate time (1–5 ms), specific of time-resolved luminescence measurements, excitation/emission slit widths (5–20 nm), and voltage of the photomultiplier tube (500–1000 V). The highest sensitivity (highest quenching of IMID over the Tb (III)-CQDs system) was obtained for a delay time of 0.1 ms, gate time of 3 ms, excitation/emission of 10/10 nm/nm, and 780 V in the photomultiplier tube. With these parameters, the blank signal (Tb (III) + CQDs) was the highest possible, which thus provided a high linear dynamic range and sensitivity for IMID determination.

**Figure 2:**
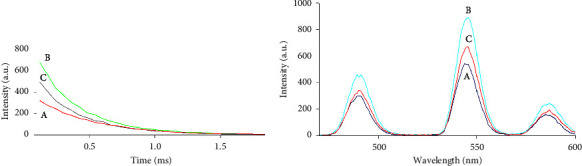
(a) Time-resolved luminescence curves of (A) Tb (III), (B) Tb (III)-CQDs, and (C) Tb (III)-CQDs-IMID solutions collected with a gate time of 5 *μ*s at excitation/emission wavelengths of 256/545 nm/nm. (b) Emission spectra of (A) Tb (III), (B) Tb (III)-CQDs, and (C) Tb (III)-CQDs-IMID solutions at an excitation wavelength of 256 nm.

Figure 2 shows the luminescence decay curves and emission spectra of Tb (III), Tb (III)-CQDs, and Tb (III)-CQDs-IMID. It can be observed that the addition of CQDs produced an increase in Tb (III) luminescence, followed by a quenching provoked by the analyte IMID.

### 3.3. Chemical Variables

The first step was the optimization of the sample treatment. In this sense, we used a commercial QuEChERS extraction kit. However, the recovery yields were low due to the pigmentation of the samples and the complex matrix of the sample. Hence, we tested the use of GCB (10–50 mg) to improve the cleaning step. The use of 40 mg GCB provided satisfactory recovery yields. IMID recoveries could decrease up to 35% when using amounts less than 40 mg of GCB. Better values were not obtained with higher amounts of GBC, so it was selected for further experiments.

The analytical parameters of the method were influenced by the pH of the solution, the concentration of terbium ions and CQDs nanoparticles, and the incubation time.

**Figure 3:**
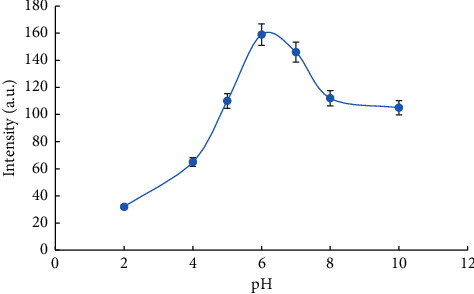
Optimization of pH value for a concentration of 1.2 mg·L^−1^ of IMID.

We optimized the pH value in the range of 2–10 (Figure 3) using solutions of NaOH and HCl to adjust the pH. For pH values of 5 and lower, the analytical signal was very low due to the protonation of the carboxylic groups present in CQDs, which prevented their interaction with Tb (III). On the other hand, pH values higher than 7–7.5 produced the hydrolysis of terbium ions, thereby decreasing the analytical signal. Therefore, the optimum pH value was 6, and different buffers were tested within this pH value. The use of Tris-HCl and phosphate buffers was tested; however, a slight decrease (10–15%) in the analytical signal was observed, and we decided to avoid the use of buffer solutions to improve the sensitivity of the system. As a result, although the pH value of the samples was close to 6 after sample treatment and dilution with ultrapure water, the dilution of the sample extracts before analysis was made with ultrapure water adjusted to pH 6 with a diluted HCl solution.

Under the optimal instrumental conditions and pH value, the concentrations of Tb (III) and CQDs were optimized (details and Figures S2 and S3 are given in Supplementary Material). Optimum concentrations of 1.5 × 10^–3^ mol·L^−1^ and 10 mg·L^−1^ were obtained for Tb (III) and CQDS, respectively. Then, the incubation time was also studied (Supplementary Material, Figure S4), obtaining the optimum signal 15 min after mixing the solutions.

### 3.4. Interference of Coexisting Foreign Substances

The main compounds that may interfere with the determination of IMID in the analyzed food samples are other pesticides. We selected malathion, glyphosate, and chlorpyrifos as general pesticides and acetamiprid and lambda-cyhalothrin as specific for the treatment of caneberries. The potential interference of some common neonicotinoids was also studied. Considering the repeatability of the method (lower than 3%), we assumed that no interference was produced by each pesticide if the variation of the signal was lower than 3%. Tolerance levels (foreign species/analyte, w/w) were 2 for acetamiprid, thiacloprid, glyphosate, and lambda-cyhalothrin and 5 for malathion and chlorpyrifos under the optimum conditions of the analytical methods. The selectivity of thiamethoxam and clothianidin (common neonicotinoids) was also studied, checking that they also interacted with the proposed system. This interaction with terbium has been previously studied in water samples by our research group; although in this case, it was required to use surfactants; however, its use is not recommended according to the principles of green chemistry.

Regarding the different tolerances observed for neonicotinoids during the interference study, it is attributed to the different chemical structures between the neonicotinoids acetamiprid and thiacloprid compared to IMID, thiamethoxam, and clothianidin (similar structures for the latter three). Although usually only one nicotinoid is used in real samples and thiamethoxam and clothianidin are not common in caneberries, chromatography-mass spectrometry would be required to confirm the exact nature of the contaminant after a positive value with the proposed system (screening method).

**Figure 4:**
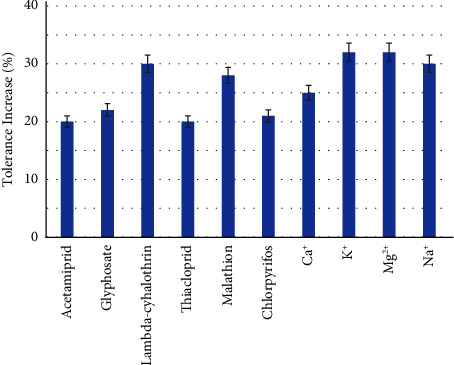
Increase (%) in tolerance ratio with the addition of CQDs (optimized conditions) compared to the absence of CQDs.

We also checked if CQDs not only improved the analytical signal, as mentioned previously, but also the selectivity of the system. Hence, we calculated the tolerance levels using only Tb (III), observing that the addition of CQDs produced an increase in selectivity between 20 and 33% for the tested interferents. Therefore, the implementation of CQDs in the system improved both the sensitivity and the selectivity of the method. In fact, the increase in selectivity is probably related not only to the interaction with CQD but also to the better sensitivity.  Figure 4 shows the increase of tolerance achieved by the addition of CQDs.

As the analyzed samples are reported to present a high content of minerals, we also studied the potential interference from some common minerals. We obtained tolerance levels higher than 100 (which was the maximum ratio tested) for Ca^2+^, K^+^, Mg^2+^, and Na^+^.

### 3.5. Figures of Merit

The analytical parameters of the proposed method were carefully evaluated under the optimized conditions previously established. They are summarised in Table 1.

Net analytical signal (quenching produced by IMID) was represented *vs.* concentration in order to obtain the corresponding calibration graph. All solutions were measured in triplicate to calculate the standard deviation (SD) in all cases. The proposed method presented a good linear relationship (*r* = 0.9994) between the quenched luminescence intensity and IMID concentration over the range of 0.1–2.5 *μ*g·mL^−1^. This linear dynamic range is lower than the expected one in luminescence systems; however, this is a common issue in analytical methods based on the quenching of luminescence [8, 9]. The instrumental limits of detection (LOD) and quantification (LOQ) were evaluated as the IMID concentration produced an analytical signal equal to 3 and 10 times the SD of the background luminescence, respectively. The obtained LOD and LOQ were 0.03 *μ*g·mL^−1^ and 0.1 *μ*g·mL^−1^, respectively. This LOD was 30% lower when compared to the obtained value by using Tb(III) with no CQDs addition. In addition, we report in Table 1 the detection limit and linear dynamic range in real samples (directly in mg·kg^−1^), after considering the sample treatment and the required dilution to analyze the samples avoiding any matrix effect. The MDL obtained in caneberries (directly in the solid samples, considering both sample treatment and dilution) was 0.9 mg·kg^−1^ IMID. This value fulfills the MRL established in Codex Alimentarius for this kind of fruit [26].

Ten independent analyses of IMID solutions, with concentrations of 0.2 and 1 *μ*g·mL^−1^, were analyzed to establish the repeatability of the method, making all the analyses within the same day. Intermediate precision was evaluated for five consecutive days, using IMID concentrations of 0.4 and 1.4 *μ*g·mL^−1^. Relative standard deviations (RSD) lower or equal to 5% were obtained in all cases.

Finally, several instrumental and chemical variables were slightly modified to study the robustness of the system. Specifically, variations of ±5% were introduced in the next parameters: photomultiplier tube voltage, measurement wavelengths (excitation and emission), and Tb (III) and CQDs concentrations. Variations lower than 4% were obtained in all cases when compared with the optimal conditions established.

The analytical parameters of the proposed method have been compared with other methods to determine IMID in food samples (Table 2). Although the LOD of the proposed method is higher than in other works, it presents several advantages for the specific samples analyzed here. First, this is the first spectroscopic method for the analysis of IMID in caneberries, and even though the LOD is not particularly low, it complies with the MRL established by Codex Alimentarius. On the other hand, the precision and recovery yields of the proposed method are similar or better than in other works. Finally, the reagents required are all commercially available and the method is simple and quick. For these reasons, the proposed method can be used for screening purposes.

### 3.6. Analytical Applications

Caneberries include several familiar berries, such as blueberries, raspberries, blackberries, loganberries, boysenberries, marionberries, and mulberries. These fruits have high levels of phenolic compounds, such as anthocyanins, as well as ascorbic acid and minerals; hence, their consumption in a normal diet presents health benefits, such as the reduced risk of cardiovascular diseases and type 2 diabetes, cancer prevention effects, and benefits in gut health and microbiota [37, 38]. We selected some samples of caneberries, specifically blackberries, raspberries, blueberries, and mulberries, to check the applicability of the method for the analysis of IMID in food samples.

An initial analysis of all samples was performed to confirm the absence of IMID in the samples. Then, potential matrix effects were evaluated by preparing IMID standard solutions in blank extracts (of each of the selected berries) by external calibration and standard addition methodology. We did not observe matrix effects in any of the samples, so external calibration was used in further experiments.

For recovery experiments, samples were spiked with appropriate IMID concentrations (adding the required volume of the IMID stock solution of 20 mg L^−1^ to 10 mg of the sample) before sample treatment (detailed in Section 2.4). They were thoroughly homogenized and kept in the dark for at least 2 hours before sample treatment. To select the spike concentrations, we considered the MRL of IMID in these kinds of samples. According to Codex Alimentarius, the MRL for berries and other small fruits (except for cranberries, grapes, and strawberries) is 5 mg kg^−1^. Hence, we spiked the samples at levels close to this MRL. The results are summarised in Table 3, which show recovery yields between 96% and 104% and RSDs lower or equal to 4% in all samples. Samples were spiked before sample treatment, and each sample was independently spiked and analyzed in triplicate. Hence, the presented data include both the recovery yields and the precision of the method (including the sample treatment step).

## 4. Conclusions

In this work, we have developed an analytical method based on CQDs-Tb (III) for the determination of imidacloprid in food samples. Although this pesticide was banned by the European Union in 2018, it is still used in many countries, and it is one of the few pesticides regulated in caneberries. It is hence important to develop rapid and accurate analytical methods for its determination to evaluate the food safety or potential risks to consumer health. In the proposed method, we have used a time-resolved luminescence probe based on CQDs-Tb (III). The advantages of the long decay time of lanthanide ions (which permits to eliminate fluorescence background signals) were coupled with the inherent advantages of CQDs. As a result, the sensitivity and selectivity of the systems were improved, and the proposed method fulfills the MRL established for IMID in caneberries. In addition, we selected commercial NPs, so the experiments could be replicated in other laboratories (a handicap of lab-made NPs is the difficulty of replicating experiments interlaboratories). The recovery experiments provided yields close to 100%, demonstrating that this method could represent an alternative to other existing methods for quality control of foods involving the potential presence of IMID.

## Figures and Tables

**Table 1:** Analytical parameters.

Parameters	
Excitation/emission slits (nm/nm)	10/10
Photomultiplier tube voltage (V)	780
*Calibration graph*	
Intercept	5.678
Slope (mL·*μ*g^−1^)	129.5
Correlation coefficient	0.9994
Linear dynamic range (*μ*g·mL^−1^)	0.1–2.5
LOD (*μ*g·mL^−1^)	0.03
LOQ (*μ*g·mL^−1^)	0.1
MDL (mg·kg^−1^)	0.9
Linear dynamic range (mg·kg^−1^)	3–75
*Repeatability (%)*	
0.2 *μ*g·mL^−1^	2.7
1 *μ*g·mL^−1^	2.3
*Intermediate precision (%)*	
0.4 *μ*g·mL^−1^	4.8
1.4 *μ*g·mL^−1^	4.5

**Table 2:** Comparison of analytical parameters with alternative methods for the analysis of IMID.

Method	Sample	Sample treatment	LOD (mg·kg^−1^)	Recovery (%)	RSD (%)	Ref.
UHPLC-MS/MS	Barberry	QuEChERS	0.005	88–90	12	[13]
UHPLC-MS/MS	Honey, pollen, royal jelly	QuEChERS, d-SPE or DLLME	0.1–3	80–109	<10	[14]
HPLC-MS/MS	Milk	d-SPE	0.13	87–96	<5	[16]
SERS	Green textile materials		3 (ng)		7	[18]
SERS	Mango fruits	QuEChers	0.02		13	[19]
Chemiluminescence	Test strips	Reaction with luminol	1.7 (*μ*g·L^−1^)	83–103	<10	[20]
Chemilum-ELISA	Honeybee	LLE	0.11 (*μ*g·L^−1^)	73–116	<9	[21]
Fluorescence	Bok choy		1.67 (mg·L^−1^)	94–100	3	[22]
Fluoroimmunoassay	Rice, apple	Gold nanoclusters	0.1 (*μ*g·L^−1^)	85–107	<6	[23]
Fluorescence	Soil, orange, tomato	MIP	0.011 (*μ*g·L^−1^)	95–101	1.2	[24]
Fluorescence	Fruits and vegetables	Upconversion nanomaterials	0.032 (*μ*g·L^−1^)	83–115	<9	[25]
Proposed	Caneberries	QuEChers	0.9	96–104	<5	

SERS, surface-enhanced Raman spectroscopy; d-SPE, dispersive solid-phase extraction; DLLME, dispersive liquid-liquid microextraction; LLE, liquid-liquid extraction; MIP, molecular imprinted polymer; LOD, limit of detection; RSD, relative standard deviation.

**Table 3:** Recovery experiments performed on berry fruits.

Sample	Added (mg·kg^−1^)	Recovery ± RSD (%)
Blackberries-1	3	99 ± 1
	5	101 ± 2
	10	103 ± 3

Blackberries-2	5	102 ± 1
	10	102 ± 3
	15	104 ± 4

Blackberries-3	3	99 ± 2
	10	100 ± 1
	20	98 ± 2

Raspberries-1	3	102 ± 1
	6	101 ± 3
	12	100 ± 4

Raspberries-2	5	103 ± 2
	10	104 ± 2
	20	101 ± 3

Raspberries-3	5	103 ± 2
	15	104 ± 2
	20	101 ± 3

Blueberries-1	3	99 ± 1
	5	96 ± 2
	10	99 ± 1

Blueberries-2	4	99 ± 3
	9	96 ± 1
	15	98 ± 2

Mulberries-1	3	97 ± 3
	7	98 ± 1
	13	101 ± 3

Mulberries-2	4	97 ± 2
	10	99 ± 3
	20	97 ± 4

## Data Availability

The data used to support the findings of this study are included within the article.

## Supplementary Materials

Supplementary Materials File S1: sample treatment. File S2: chemical variables. Figure S1: Stern–Volmer plot for the CQDs-Tb (III)-IMID system. Figure S2: optimization of Tb (III) concentration: 10 mg·L^−1^ of CQDs and 1.2 mg·L^−1^ of IMID. Figure S3: optimization of CQDs concentration: 1.5 × 10^–3^ mol·L^−1^ of Tb (III) and 1.2 mg·L^−1^ of IMID. Figure S4: evaluation of the incubation time influence: 1.5 × 10^–3^ mol·L^−1^ of Tb (III), 10 mg·L^−1^ of CQDs, and 1.2 mg·L^−1^ of IMID.
