# Supplementary material for: Luminescent Probe Based on Terbium-Carbon Quantum Dots for the Quantification of Imidacloprid in Caneberries
Source: J Anal Methods Chem. 2023 Oct 30;2023:5561071. doi: 10.1155/2023/5561071 (PMC10627717; doi:10.1155/2023/5561071)
Supplement: Supplementary Materials — File S1: sample treatment. File S2: chemical variables. Figure S1: Stern–Volmer plot for the CQDs-Tb (III)-IMID system. Figure S2: optimization of Tb (III) concentration: 10 mg·L−1 of CQDs and 1.2 mg·L−1 of IMID. Figure S3: optimization of CQDs concentration: 1.5 × 10^–3^ mol·L−1 of Tb (III) and 1.2 mg·L−1 of IMID. Figure S4: evaluation of the incubation time influence: 1.5 × 10^–3^ mol·L−1 of Tb (III), 10 mg·L−1 of CQDs, and 1.2 mg·L−1 of IMID. [file 5561071.f1.docx]

**SUPPLEMENTARY MATERIAL**

**Luminescent probe based on terbium-carbon quantum dots for the quantification of imidacloprid in caneberries**

Eulogio J. Llorent-Martínez*, Julia Jiménez-López, Antonio Ruiz-Medina

*Department of Physical and Analytical Chemistry, Faculty of Experimental Sciences, University of Jaén, Campus Las Lagunillas, E-23071 Jaén, Spain*

**File S1: Sample treatment**

10 g of sample and 10 mL acetonitrile were mixed in a 50 mL PTFE centrifuge tube. Then, after adding the content of a 15 mL tube extraction kit (4 g MgSO_4_, 1 g Na_3_C_6_H_5_O_7_, 0.5 g Na_2_C_6_H_6_O_7_ · 1.5H_2_O and 1 g NaCl), the samples were vortexed for 1 min. After centrifugation at 4000 rpm for 5 min, 6 mL of the supernatant was transferred into a 15 mL dispersive solid phase extraction tube containing the sorbent (150 mg PSA and 900 mg MgSO_4_) and 40 mg of GCB. Samples were vortexed for 1 min, centrifuged (5 min, 4000 rpm) and the supernatant was diluted with ultrapure water (previously adjusted to pH 6 with HCl) before analysis.

**File S2: Chemical variables**

To optimize Tb(III) concentration (**Fig. S2**), we carried out two parallel studies (without CQDs and at a fixed CQDs concentration of 10 mg L^-1^). In both cases, Tb(III) concentration was varied between 0.5 and 10x10^-3^ mol L^-1^, obtaining similar results: the best Tb(III) concentration was 1.5x10^-3^ mol L^-1^. Then, using this terbium concentration, CQDs levels were modified between 2.5 and 50 mg L^-1^ (**Fig. S3**), establishing that the optimum concentration was 10 mg L^-1^. Finally, the incubation time was also studied in the range of 0-60 min, observing that the optimum signal was obtained 15 min after the mixing of solutions (**Fig. S4**). Hence, after mixing the solutions, we waited 15 min to obtain the analytical signal.


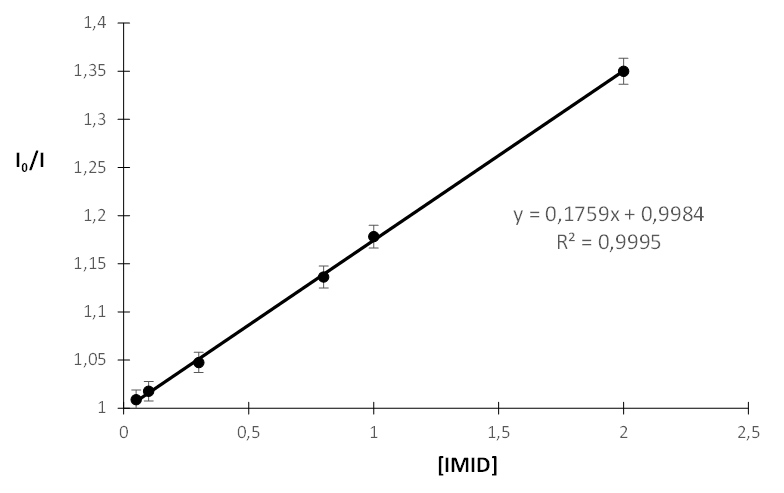


**Fig. S1**. Stern-Volmer plot for CQDs-Tb(III)-IMID system

**
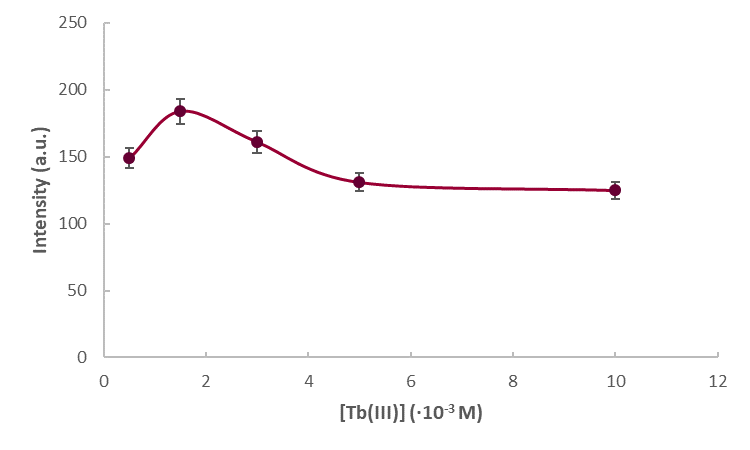
**

**Fig. S2.** Optimization of Tb(III) concentration: 10 mg L^-1^ CQDs, 1.2 mg L^-1^ IMID

**
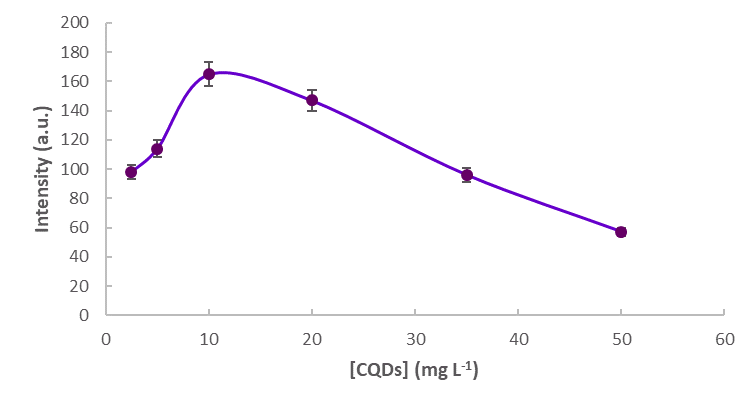
**

**Fig. S3**. Optimization of CQDs concentration: 1.5x10^-3^ mol L^-1^ Tb(III), 1.2 mg L^-1^ IMID


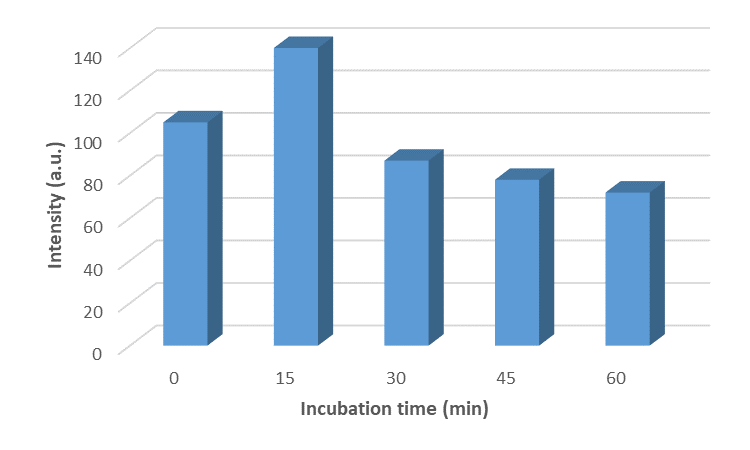


**Fig. S4**. Evaluation of the incubation time influence; 1.5x10^-3^ mol L^-1^ Tb(III), 10 mg L^-1^ CQDs, 1.2 mg L^-1^ IMID
